# A neural network model of causative actions

**DOI:** 10.3389/fnbot.2015.00004

**Published:** 2015-06-30

**Authors:** Jeremy Lee-Hand, Alistair Knott

**Affiliations:** Department of Computer Science, University of OtagoDunedin, New Zealand^†^

**Keywords:** causative actions, motor learning, event codes, neural networks, hand/arm actions, ventro-dorsal motor pathway

## Abstract

A common idea in models of action representation is that actions are represented in terms of their perceptual effects (see e.g., Prinz, 1997; Hommel et al., 2001; Sahin et al., 2007; Umiltà et al., 2008; Hommel, 2013). In this paper we extend existing models of effect-based action representations to account for a novel distinction. Some actions bring about effects that are independent events in their own right: for instance, if John *smashes* a cup, he brings about the event of *the cup smashing*. Other actions do not bring about such effects. For instance, if John *grabs* a cup, this action does not cause the cup to “do” anything: a grab action has well-defined perceptual effects, but these are not registered by the perceptual system that detects independent events involving external objects in the world. In our model, effect-based actions are implemented in several distinct neural circuits, which are organized into a hierarchy based on the complexity of their associated perceptual effects. The circuit at the top of this hierarchy is responsible for actions that bring about independently perceivable events. This circuit receives input from the perceptual module that recognizes arbitrary events taking place in the world, and learns movements that reliably cause such events. We assess our model against existing experimental observations about effect-based motor representations, and make some novel experimental predictions. We also consider the possibility that the “causative actions” circuit in our model can be identified with a motor pathway reported in other work, specializing in “functional” actions on manipulable tools (Bub et al., 2008; Binkofski and Buxbaum, 2013).

## 1. Introduction

A common idea in models of action representation is that an agent's actions are encoded in a way which makes reference to the sensory effects they bring about. This idea has a long history, but in recent research it is most strongly associated with Prinz's (1997) theory of “common coding” and Hommel et al.'s theory of “event codes” (Hommel et al., 2001). The key idea uniting these models is that motor programs are not defined purely within the motor domain: their neural representation includes a representation of the effects they are expected to have on the world, as apprehended by the perceptual system. This idea has been supported in a variety of experiments, and modeled computationally in a number of different ways, as we will summarize below.

In this paper our aim is to extend existing computational models of effect-based action representations to account for a distinction that has so far been overlooked. Some causative actions bring about effects that are independent events in their own right. For instance, if John *smashes* a cup, he brings about the event of *the cup smashing*. This is an event which in other circumstances could happen independently of any action of John's: it involves the cup changing state in a certain way. In the right circumstances, John could perceive the event of the cup smashing simply by passively observing the cup. Similarly, if John *opens* a door, he brings about the event of *the door opening*, in which the door undergoes a change of state, which in other circumstances John could perceive independently of any action of his own. On the other hand if John *touches* or *grasps* a cup, he does not bring about any event involving the cup that is independent of his own action. Crucially, touching or grasping the cup does not have to bring about any specified change of state in the cup (If there are changes of state, they are incidental to the action, rather than part of its definition). The distinction we want to highlight can be summarized as follows: “to smash a cup” means “to *cause* the cup to smash, and “to open a door” means “to *cause* the door to open. But “to touch a cup” doesn't mean “to cause the cup” to do anything (It certainly doesn't mean “to cause the cup to touch”). And “to grasp a cup” doesn't mean “to cause the cup” to do anything.

Nonetheless, actions like touching and grasping can certainly be thought of as defined by the perceptual effects they bring about, as we will discuss. In fact, a lot of the research into effect-based representations of motor actions has focussed on simple actions like touching and grasping. We must therefore conclude that there are at least two different *types* of effect-based action representation, which are structurally distinct. In this paper we will develop a model of causative actions that captures this distinction.

In the remainder of this section, we review current empirically-derived and computational models of effect-based action representations, and in the light of these, we introduce some design principles governing the new model we develop.

### 1.1. Experimental evidence for effect-based motor representations

Experimentally, the idea that actions are defined by their effects has been supported in several ways. For instance, there have been many studies exploring variations on the well-known stimulus-response compatibility effect (Simon, 1969). A good example is a study by Hommel (1993). Here subjects had to respond to an auditory stimulus by pressing a button, either with the left or right hand. The tone of the auditory stimulus indicated which button the subject should press. But as a distracting factor, the stimulus was also presented either on the left or the right. The classical stimulus-response compatibility effect is that subjects are slower to respond if the spatial location of the stimulus is incompatible with the hand which must respond. In Hommel's experiment, button presses generated a reafferent visual stimulus whose location could be decoupled from the location of the hand pressing the button, to explore whether the compatibility effect operates in the domain of motor movements or that of their sensory consequences. Button presses consistently produced a visual stimulus: illumination of a light. In one condition the light appeared on the same side as the hand (e.g., left button presses illuminated a light on the left), while in another it appeared on the opposite side (e.g., left button presses illuminated a light on the right). Hommel found that the stimulus-response compatibility effect depended on compatibility with the perceptual effects of button-presses, rather than on the hand which was used. This shows that the way subjects encode actions does make some reference to their sensory consequences—at least enough to interfere with stimulus-response mappings.

The idea of effect-based representations of motor actions is also supported by several animal studies of the neural representation of actions. For instance (Umiltà et al., 2008) observed the activity of neurons in premotor area F5 of monkeys performing grasp actions with specially constructed tongs. F5 neurons respond to a range of grasp movements made by the hand (see e.g., Rizzolatti et al., 2000). But under normal circumstances, executed grasp movements correlate strongly with visual signals. The experiment was designed to decouple motor movements from their observed effects. In one condition monkeys used a regular pair of pincers, which closed when the monkey squeezed. In another, they used reversed pincers, which opened when the monkey squeezed, and closed when they relaxed their grip. Most F5 neurons responded in the same way in both conditions: their activity was a function of the movement of the pliers rather than the movement of monkeys' hands. This is evidence that many neurons in this grasp-planning area encode the effects of motor movements, rather than their properties as motor movements.

An interesting type of experimental evidence for effect-based motor representations comes from studies showing what is termed an **ideomotor response** (see Hommel, 2013 for a review). In these studies, the subject first learns that certain actions cause certain perceptual effects, and then, after learning, is presented with these same perceptual effects as independent perceptual stimuli. These stimuli can be shown to activate representations of the actions that cause them, for instance by speeding subsequent execution of these actions, or by influencing selection of these actions (Elsner and Hommel, 2001). This triggering of action representations by representations of their perceptual effects is the so-called ideomotor response. Evidence for the ideomotor response shows not only that representations of actions include reference to their perceptual effects, but also that activation of the associated perceptual effects can actually cause activity in the action preparation system. The ideomotor response provides a useful tool for studying the neural structures involved in linking actions to their perceptual effects. Elsner et al. (2002) interleaved action effect stimuli in different proportions with neutral perceptual stimuli. They found that activity in the supplementary motor area (SMA) and right hippocampus correlated with these proportions, suggesting that these areas are involved in the circuit that maps perceived effects onto motor programs. Melcher et al. (2013) investigated the process by which the ideomotor response was learned. They examined brain activity over time in two groups of subjects: an experimental group, whose actions generated consistent perceptual effects, and a control group where there were no consistent perceptual effects. They looked for brain areas where the ideomotor response increased as a function of time during learning in the experimental group but not in the control group. They found such effects in the hippocampus, parahippocampal gyrus, caudate nucleus, and angular gyrus, suggesting that these areas are additional components of the circuit.

A final perspective on effect-based action representations comes from studies of the neural circuits subserving the use of tools. In recent research, evidence has emerged that there are two distinct pathways in the dorsal circuit that maps visual representations onto motor actions: a “dorso-dorsal” pathway generates visually guided reach/grasp actions on a target object, while a “ventro-dorsal” pathway, sometimes called the “use” pathway, generates actions that manipulate a target object in accordance with its conventional use, to achieve particular effects (see e.g., Bub et al., 2008; Binkofski and Buxbaum, 2013). These pathways were discovered in neuroanatomical studies of macaque (see e.g., Rizzolatti and Matelli, 2003): the dorso-dorsal pathway runs through areas V6 and MIP in the superior parietal lobule to dorsal premotor cortex, while the ventro-dorsal pathway runs from superior temporal cortex (MT/MST/STP) through the inferior parietal lobule (AIP/VIP) to the ventral premotor cortex. Damage to the two pathways results in distinct patterns of dysfunction, in both macaques and humans: damage to the dorso-dorsal pathway results in optic ataxia, a deficit in visually guided reaching and grasping, while damage to the latter pathway leads to ideomotor apraxia, a deficit in the ability to generate functionally appropriate actions on tools (especially if the tools are not visually present). From these deficits, a model emerges in which the dorso-dorsal pathway maps visually derived “volumetric” representations of the location, orientation, and shape of target objects onto suitable reach/grasp actions, while the ventro-dorsal pathway maps internal representations of object categories onto functionally appropriate actions^1^. In this model, for example, the dorso-dorsal pathway would map a “volumetric” visual representation of a stapler onto the action required to reach it or pick it up, while the ventro-dorsal pathway would link an internal representation of the object category “stapler” to the specialized manipulatory action that causes a stapler to perform its function of stapling (The representation of object category can be generated either perceptually, through the ventral pathway for object classification, or alternatively via language: see e.g., Masson et al., 2008; Bub and Masson, 2010; Jax and Buxbaum, 2010; Binkofski and Buxbaum, 2013). Exactly how the ventro-dorsal pathway learns such affordances is still a matter for investigation; however, it is clear that action effects are of central relevance: the agent must learn the actions that cause the tool to behave in the way it was designed to—for instance, the action that causes a stapler to staple.

In summary, there is good evidence that agents represent motor actions in terms of their effects, and that representations of perceived effects have a functional role in generating actions. We also know something about the brain circuits that implement associations between actions and their effects—and in particular, we know there is a circuit that specializes in manipulatory actions that achieve functional effects on objects of particular categories^2^.

### 1.2. Computational models of effect-based action representations

The experiments reviewed above do not provide detailed information about the nature of effect-based action representations, or about the mechanisms through which they are learned and triggered. These topics are currently most amenable to study in computational simulations of perceptuomotor learning in robots (for reviews see e.g., Weng et al., 2001; Asada et al., 2009). There have been several computational models of how effect-based action representations develop; in this section we will review some key themes, both within robotics models and in models that explicitly simulate neural processes.

In robotics models, the basic idea that actions are defined by their perceptual effects is often expressed within a model of Gibsonian affordances. For instance (Sahin et al., 2007) define an affordance as a relation linking an “action” (a motor representation) plus a “target” (a perceptual representation of an external entity) with an “effect” (a perceptual representation of a change in the external entity), and refer explicitly to Elsner and Hommel (2001). A similar scheme is adopted by Stoytchev (2008), in a system that learns to associate actions on target objects with perceptual changes to these objects. Various different types of action effect have been explored, including translational movements of the agent (Dogar et al., 2007) or of the target (e.g., Stoytchev, 2008; Ugur et al., 2011) and changes to the configuration of an articulated target (e.g., Katz and Brock, 2008).

In neurally-inspired computational models of motor control and motor learning, there are several explicit models of the role of perceptual effects. Tactile effects play an important role in Oztop et al.'s (2004) model of infant grasp learning: in this model, touch sensations are inherently rewarding, and focus the system's learning on actions that result in touch sensations. Arbib et al. (2009) discuss how this model can be extended to account for the process by which an agent learns to use a tool, with particular focus on the results of Umiltà et al. (2008) discussed earlier. In this model, an important idea is that when learning to use a tool, an agent initially uses an “effector-independent” representation of his/her current task goal to control movements (using perceptual feedback to ensure the goal is achieved), and progressively learns a tool-specific forward model to achieve the same results. An effector-independent representation is a perceptual representation, so this model certainly accounts for how an agent learns to associate motor programmes with perceptual effects, although its focus is on the idea that tool-specific forward models can be regarded as extensions of the agent's body schema. Finally, action effects are also considered in the TRoPICALS model of Cagliore et al. (2010). This model also uses touch sensations to control learning of reach and grasp actions: during training, the function mapping a visual representation of the target to the current motor state is learned when the agent's fingers are touching the target. TRoPICALS does not simulate manipulatory actions on objects, but it does include a particularly detailed model of the visual pathways that generate representations of the location and shape of visual stimuli, and of the visual pathway running though ventral/inferotemporal cortex to prefrontal cortex, that maps the shape of a perceived object onto a semantic category representation, and then to a representation of associated action goals, which we will refer to when discussing our own model.

Many of the above models construe action-effect learning within a sequence of developmental stages. For instance, Metta and Fitzpatrick's model (Metta and Fitzpatrick, 2003) envisages three stages. In the first stage, the agent learns about the immediate visual effects of its own actions: in particular, to recognize its moving arm as a visual stimulus that is predictable from its own motor movements. In the second stage, the agent learns simple visual representations of external objects by bumping into them with its arm: a visual stimulus representing an object is defined as a stimulus that predictably changes when the arm (also represented visually) arrives in its vicinity. In the third stage, the agent learns more complex object representations, which are linked to particular grasp/manipulation actions that have their own visual characteristics and can potentially be identified in other agents. Similar notions of developmental stages are proposed by other researchers (e.g., Dogar et al., 2007, building on the formalization of Sahin et al., 2007; Stoytchev, 2009). Oztop et al's (2004) model also envisages two developmental stages: one for learning reach actions, and another, building on this, for learning grasp actions.

However, the development of causative actions is characterized, an interesting observation is that complex causative actions tend to incorporate, or make reference to, simpler causative actions acquired earlier in development. The case of touching/grasping actions is particularly relevant. In order to achieve an effect on an external object, the agent must first make contact with this object (In fact most causative actions incorporate some kind of touch or grasp, though we will consider some exceptions in Section 4). Some models, such as that of Arbib et al. (2009), focus on manipulatory actions that take place once a stable grasp has been established on the target object. Other models consider the actions that biring the hand into contact with the object; this is most explicit in the model of Stoytchev (2008). Stoytchev defines two components of a motor action: one is a **binding behavior**, that brings the effector into some kind of contact with the target object (possibly a stable grasp); the other is an **exploratory behavior** that takes place after binding, that can achieve effects on the target. However, in Stoytchev's experiments, binding behaviors and exploratory behaviors occur in strict sequence: the robot first achieves a stable grasp on a target object and then explores ways to achieve effects on it. This approach does not work for all causative actions: sometimes, to achieve a particular effect on a target object, the hand must achieve contact in a particular way. For instance, to cause an object to break or deform, the hand needs to approach it with a certain force, and perhaps from a certain direction. So while a breaking action certainly involves a touching action, it may be quite different from the touch action learned early in development as an action in its own right. We will return to this point below.

A final theme in current computational models of action-effect learning is the idea that the agent must learn *categories* of perceptual effect before it can start learning how actions relate to effects. Recall that what the agent is learning is *categories* of action; several computational models express the idea that these action categories have their origin in perceptual categories. This idea leads to another notion of developmental stages. In an initial stage, the agent learns categories of perceptual stimulus that result from exploratory motor interactions with its environment. These categories are learned using unsupervised methods, often involving clustering and/or self-organizing maps (see e.g., Griffith et al., 2011; Ugur et al., 2011). In a subsequent stage, the actions that generate stimuli from these perceptual categories are learned. The learning of all effect-based actions, whether simple or complex, is best thought of as involving these two distinct sub-stages. In the TRoPICALS model of Cagliore et al. (2010), unsupervised methods for learning associations are also used, in learning a mapping from visual features to object categories in the ventral pathway, and for learning higher-level correlations in the prefrontal cortex between object categories and task in structions.

### 1.3. Design principles for a model of causative actions

A key design principle for our motor model relates to the distinction discussed at the start, between two kinds of action that can be performed on a target object. To recap: there are some actions whose perceptual effects are events involving the target object that can take place independently in the world without any intervention by the agent, and there are others whose perceptual effects are not independent in this way. Actions of the former kind include smashing, opening, bending and squashing: “to smash/open/bend/squash X” means “to cause X to smash/open/bend/squash.” We will call actions of this kind **causative**. Actions of the latter kind include touching, grabbing, slapping, and punching: “to touch/grab/slap/punch X” does not mean “to cause X to ϕ,” for any action ϕ. Since these actions cannot be represented using this explicitly causative template, we will call them **non-causative**. At the same time, these latter actions can still undoubtedly be defined in terms of their effects: in fact in the developmental models of Metta and Fitzpatrick (2003) and Stoytchev (2009), actions like touching and grabbing are among the earliest effect-based actions to be learned. Our first proposal is that causative and non-causative actions are implemented in *separate networks* in the motor system, that obtain their information about action effects from different perceptual modules. We make specific proposals about the perceptual modules involved in each case. We propose the network that implements causative actions gets its representations of action effects from the same perceptual module that is used to identify independent events taking place in the world: the network that recognizes changes taking place in external objects (Beck et al., 2001; Donner et al., 2007), or actions being performed by external agents (e.g., Grafton and Hamilton, 2007; Chong et al., 2008). In our model, this network, which operates by itself when the agent is passively perceiving the world, is also recruited for a role in training the causative actions network. We propose that the network implementing non-causative actions gets its effect representations from a different perceptual module, namely the *tactile* system. The example non-causative actions introduced above (touching, grabbing, slapping, and punching) are all actions whose execution results in a well-defined tactile stimulus. Touching is an action which results in *any* tactile stimulus. Grasping, slapping and punching all result in distinctive tactile stimuli, which arguably fall into well-separated perceptual classes: a grasp results in stable contact within an opposition surface of the hand, a slap results in transitory contact with the open palm, a punch results in forceful contact with the knuckles. While the models of effect-based action representations reviewed in Section 1.1 focus on visual effects, our model will highlight the role of tactile perceptual representations in the learning of non-causative action categories.

A second proposal relates to the idea that effect-based action representations are acquired in a developmental trajectory, as already discussed above. In our model, the network implementing causative actions is an *extension* of that implementing non-causative actions. In fact we also assume that there are two separate networks implementing non-causative actions: a basic network implementing the “touch” action, and a second network built on top of this network implementing the other non-causative actions. This assumption is commonly accepted; there is good evidence that the network implementing “grasp” actions is an extension of the network implementing simple reach actions (see e.g., Jeannerod, 1996). In summary, we envisage that the mechanism for learning effect-based motor actions includes three distinct sub-networks, that operate on increasingly complex perceptual representations, and are trained at successive developmental stages: one controlling reach-to-touch actions, one controlling simple hand actions, and one controlling causative actions.

A final proposal in our model is that each of these networks implements similar computations. In particular, we envisage a core computation replicated in each network, in which a perceptual signal, representing the perceptual outcome of an experimentally executed action, is *copied* into a motor medium, representing a simple or complex motor goal. This operation allows a clear distinction between *actual* perceptual stimuli and *intended* perceptual stimuli. A perceptual stimulus should not always trigger a motor action. It reliably generates *some* motor activity, as shown in experiments demonstrating the ideomotor response. But this activity is often best viewed as a side-effect of the agent's learning, rather than as something functional. It is only when the agent activates a representation of a perceptual effect *as a goal* that it should trigger an actual motor action. The core circuit in each of our networks represents perceptual stimuli as they are observed and as they are intended in separate media, to enforce this distinction. However, these media are linked by 1:1 connections, which allow copying of perceptual representations into the motor system under specific circumstances during motor learning. In our model it is these copy operations that implement the principle that actions are defined by their perceived effects.

An important possibility to consider is that the distinction we propose between networks for causative and non-causative actions *coincides with* the distinction made in earlier work between a dorso-dorsal “reach/grasp” pathway and a ventro-dorsal “use” pathway. There are certainly obvious similarities: the dorso-dorsal pathway produces reach/grasp actions, like our network for non-causative actions, while the ventro-dorsal pathway produces actions on tools, which often conform to our criteria for causative actions. For instance, the tool-use actions defined as “functional” in Jax and Buxbaum's (2010) experiment are nearly all causative in our terms: “using” a stapler involves causing it to staple, “using” a hole punch involves causing it to punch, “using” a toaster involves causing it to toast, “using” a pump involves causing it to pump, “using” a padlock involves causing it to lock or unlock. The same is true for Bub et al.'s (2008) “functional” actions: for instance, ringing a bell involves causing it to ring; firing a water pistol involves causing it to fire. There are some exceptions—for instance, Bub et al. classify grasping a beer mug by its handle as a functional action, but this action doesn't cause the mug to do anything—but the correspondence is quite close. Conversely, the “volumetric” actions assumed to engage the dorso-dorsal pathway in Bub et al.'s experiment are typically non-causative actions by our criteria: for instance, a power grasp on a bottle or a precision grasp on a paintbrush don't cause the target object to do anything. Again the correspondence is not perfect: for instance, Bub et al. assume that actions which move an object are controlled in the dorso-dorsal pathway, but by our criteria such actions would be causative (“moving an object” involves causing it to move), and there are many actions that are non-causative by our criteria that have not been used in experiments as examples of volumetric actions (for instance actions of slapping or punching a target object). Nonetheless, there is enough of an overlap to raise the possibility that the distinction between actions in the “reach/grasp” and “use” dorsal pathways is really a distinction between causative and non-causative actions, and is best characterized in these terms. In fact, this idea also sits comfortably with our proposal that the effects of causative actions are delivered by a perceptual module that recognizes independent events taking place in the world. The ventro-dorsal network includes areas in the superior temporal cortex that are involved in classifying the movements of observed external objects, of both biological and non-biological kinds (see e.g., Perrett et al., 1989; Beauchamp et al., 2002), and also inferior parietal areas participating in the “ventral attentional network” through which an agent's attention is oriented to salient stimuli in the external world (Corbetta and Shulman, 2002). Based on these considerations, we will tentatively localize the causative-actions and non-causative-actions networks in the ventro-dorsal and dorso-dorsal streams, respectively. This has several implications. Theoretically, it means that we are proposing a particular computational model of how actions are learned in these two pathways. The model of learning emphasizes the role of perceptual effects in each pathway, in accordance with the accounts of Hommel and colleagues, but also offers a new suggestion about the difference between the two pathways: actions in the dorso-dorsal network are associated with effects registered by touch, while actions in the ventro-dorsal network are associated with effects registered by the perceptual system that recognizes external events in the world. Our proposal also has experimental implications: it predicts that distinctions between actions in the “reach/grasp” and “use” pathways will emerge more sharply if they are defined using our metric for causality. Specifically, we predict that actions in the “use” pathway are actions which cause the occurrence of an independently observable event in the world, while actions in the “reach/grasp” pathway are actions which cause a characteristic tactile sensation. We will review these predictions in Section 4.

## 2. Methods

### 2.1. Software platform

**Figure 1:**
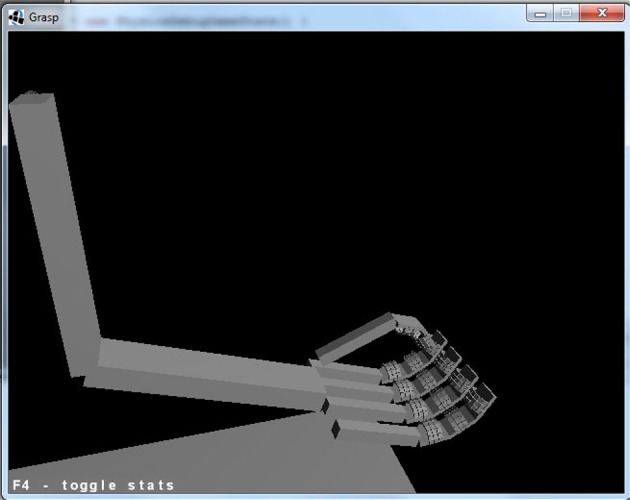
**The GraspProject environment**. Shown is the arm model that is used in our simulations.

Our simulations were conducted using the GraspProject environment (Neumegen, 2013). The environment includes a model of an articulated hand/arm, controlled by the Bullet physics engine (Bullet, 2012), and a simulated retina, on which images of the arm and target objects are rendered using (OpenGL, 2012). The hand/arm as viewed by the retina is shown in Figure 1. The arm has 3° of freedom: an elbow joint, and a ball and socket joint in the shoulder. The fingers and wrist are also flexible; the fingers include a novel implementation of soft fingerpads that are capable of detecting pressure at different locations on the hand, and distinguishing different patterns of tactile contact (for details see Lee-Hand et al., 2012). The hand also includes less sensitive sensors of touch on the back of the hand and on the palm.

**Figure 2:**
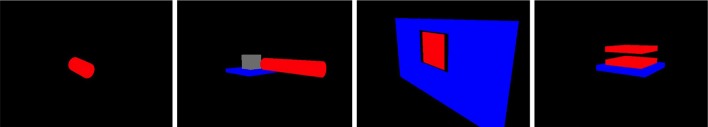
**Objects created for the simulations**. From left: a cylinder (for touching, grasping, punching, and slapping); a lever (for bending); a door (for opening); a compressible object (for squashing).

We built several objects in the simulation environment which could serve as targets for hand actions. One is a simple object (a cylinder) which serves as the target for the simple motor actions of touching, grasping, punching, and slapping. The other three are articulated objects which can undergo various changes in internal configuration, similar to the objects in Katz and Brock (2008). One is a lever which can pivot around a joint, and can be bent; one is a hinged door in a plane, which can be pushed open; one is a pair of horizontal plates connected by a spring, which can be “squashed” by pushing down on the top plate. These objects are illustrated in Figure 2, again as viewed by the retina.

### 2.2. Motor control without preplanned trajectories

In our model we adopt a particular conception of motor control, which does not bear directly on our account of causative actions, but is important to introduce, as it differs from the traditional conception of motor control.

Traditional models of motor control envisage two quite separate types of motor learning. One is the learning of a general motor control function; the other is the learning of a set of trajectories associated with particular action categories. A motor controller is traditionally (and conveniently) modeled as a general function that maps the current motor state at time *t* and the goal motor state at time *t* + *i* onto a motor command at *t* which will lead to the goal state. The motor controller is given a trajectory, represented as a precomputed sequence of goal states, and generates a sequence of commands that move the effector along this trajectory (see e.g., Jordan and Wolpert, 2000). However, there is good evidence that in humans and other biological agents, preparing an action does not involve advance computation of a detailed motor trajectory (see e.g., Cisek Cisek and Kalaska, 2005). This requires a considerable revision to the traditional conception of motor control. If motor trajectories are not fully precomputed, they must be generated “online,” while actions are under way—in other words, they must be generated *within* the system that effects motor control, rather than separately from it.

Online motor control is known to involve a mixture of feedforward and feedback control (Kawato et al., 1987). A feedback controller takes the current motor state and the goal motor state and generates a motor signal proportional to the difference between them, in a direction which reduces this difference; it does not need to be trained. A feedforward controller develops through motor learning. In a traditional model, with precomputed trajectories, a feedforward controller has a relatively circumscribed role: it simply learns about the properties of the controlled motor plant, so it can generate commands that more accurately produce the next motor state in the trajectory. In our model we broaden the concept of a feedforward controller to give it a role in learning the motor trajectories associated with different kinds of action, as well as in modifying the commands delivered by a feedback controller. In our model, the feedback controller takes a single goal motor state, associated with the end-point of an action, rather than a sequence of precomputed goal motor states. We propose that the learned, feedforward aspects of motor control that represent action trajectories are delivered by a system that generates scheduled *transformations* of this goal state, which deviate the motor plant from the normal course it would take under simple feedback control. We call these transformations “perturbations.” For instance, to generate a trajectory bringing the hand onto the target from above, the goal state could be temporarily perturbed to a point above the target, so the hand initially moves higher than it would normally do. In this view, the system that learns to supplement a simple feedback motor controller is also the system that learns action trajectories. This approach to motor motor control is described in more detail and evaluated in Lee-Hand et al. (2012). We adopt it in our current model of causative actions, although it is not an essential component of the model, and could easily be replaced by a more traditional model of motor control, involving precomputed trajectories.

### 2.3. Architecture of the motor control network

**Figure 3:**
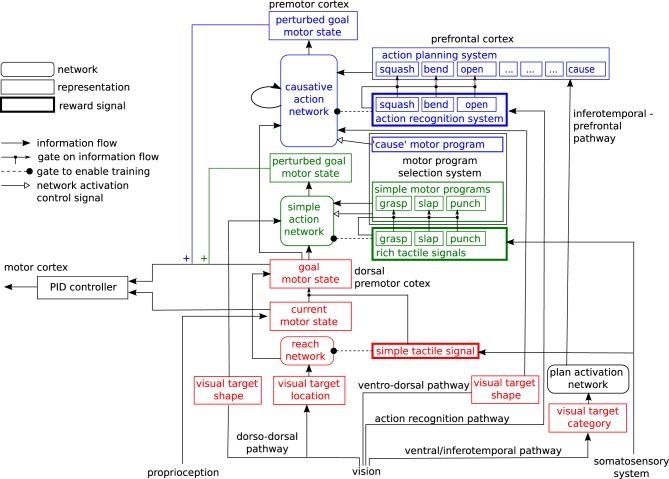
**Architecture of the motor control network**.

Our model of the motor system is expressed as a neural network for learning hand actions directed at target objects. It provides a simple model of some aspects of infant motor development. The general architecture of the network is shown in Figure 3. It consists of three sub-networks arranged in sequence, which are trained at three successive developmental stages, by reward signals of different degrees of complexity: a **reach network** (shown in red), a **simple action network** (shown in green) and a **causative action network** (shown in blue). We will describe these networks in turn (Details of their training methods are given in Appendix).

#### 2.3.1. The reach network

**Figure 4:**
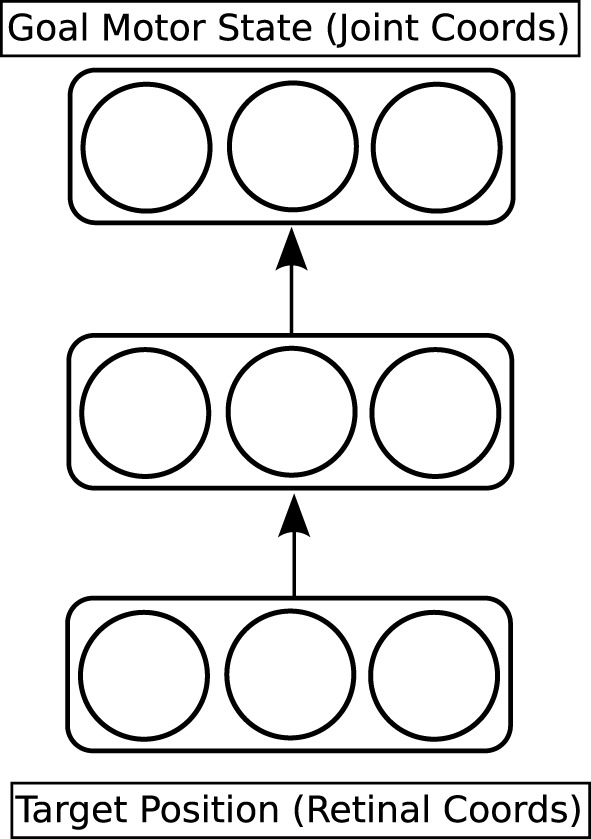
**Detailed architecture of the reach network**.

The first network to be trained is called the reach network (see the red part of Figure 3, and Figure 4). This network provides a model of the dorso-dorsal visuomotor pathway discussed in Section 1.1. It learns a function which maps a visual representation of the location of the target object onto a goal motor state of the hand and arm. The visual representation is three-dimensional. Two dimensions come from the centroid of the projection of the target object onto the simulated retina; the third dimension (depth) is read directly from the physics engine. The goal motor state is also three-dimensional: one dimension for each joint angle of the arm. The network has a hidden layer with three units, which is fully connected to both input and output layers; it is trained through back-propagation (Rumelhart et al., 1986).

During training, a target object (the cylinder illustrated in Figure 2) is placed in the hand's perispace and the agent executes hand/arm actions at random, by activating random goal motor states of the hand/arm and using a simple feedback controller (shown on the left in Figure 3) to drive the hand/arm toward these states. Sometimes the ensuing action results in the hand touching the target, evoking a touch signal (the simple touch signal). This signal is intrinsically rewarding (as in Oztop et al., 2004). The touch signal triggers two operations. First, it causes a proprioceptive representation of the agent's current motor state to be copied into the medium holding its goal motor state (see the gating link terminating on the connection between the current and goal motor states in Figure 3). Second, it causes the reach network to be trained, so that the current visual representation of the target object is associated with this newly specified goal motor state, and similar presentations of the target in the future will automatically elicit an appropriate motor goal (see the gating link terminating on the reach network).

This simple circuit implements a particular version of Hommel et al.'s model of event codes. Learning in the circuit creates what can be thought of as a single simple action category, associated with the sensory representation of a touch to the hand: after training, when the reach network is presented with the visual location of a target object, it will activate a motor goal which when achieved will reliably elicit this sensory representation. Motor goals in the circuit are associated with sensory stimuli in three ways. Any representation in the motor goal medium is implicitly associated with one particular reward stimulus (a simple touch sensation). Specific motor goals are associated axiomatically with specific motor states (sensed proprioceptively) when the reward stimulus is evoked. And specific motor goals are also associated through learning with arbitrary sensory stimuli (in this case visual), which carry information about the motor states associated with reward signals. Again this happens at the time the reward stimulus is evoked. The key devices in the circuit are reward-gated copy and learning operations. These devices are replicated in the other two networks. After learning in the reach network, the agent can reliably map the retinal coordinates of a target object onto a goal motor state in which the hand is touching the target object. Once this goal motor state is generated, it is passed as input to the simple feedback motor controller, which produces an action delivering this goal state. The feedback controller takes the current motor state (derived from proprioception) and the goal motor state (delivered by the reach network from visual input) and generates a sequence of motor signals which progressively reduce the difference between them to zero. We use a PID controller (see e.g., Araki, 2006) as our implementation of a feedback controller.

The reach network essentially learns to solve the inverse kinematics problem. In the general case, this problem has many solutions, but since our hand/arm has only 3° of freedom there is only one solution, so the network implements a simple function. However, the networks introduced in the following sections incorporate methods for choosing between alternative solutions; these methods could be adopted in the reach network to configure it for more complex inverse kinematics scenarios.

#### 2.3.2. The simple action network

**Figure 5:**
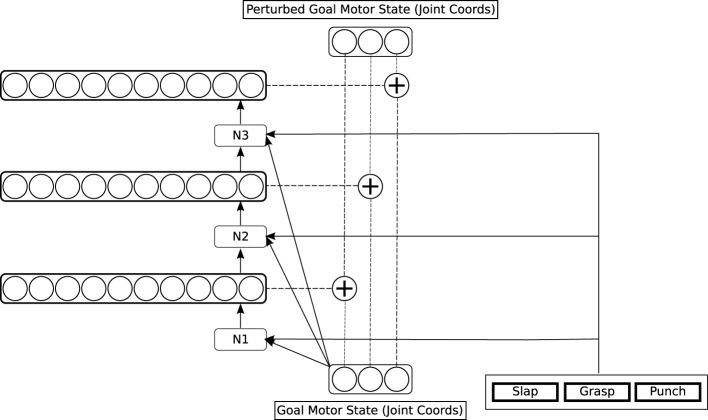
**Detailed architecture of the simple action network**.

The second network in our architecture is the simple action network (see the green part of Figure 3, and Figure 5). This network also models computations in the dorso-dorsal visuomotor pathway (Recall that this pathway controls hand preshape actions as well as arm movements. See e.g., Fattori et al., 2010 for evidence for specific areas in this pathway that represent hand shapes). The simple action network learns simple categories of action to execute on a target object, that are defined by characteristic tactile effects: these include grasping, but also actions like punching and slapping. The effects are brought about by bringing the hand/fingers into contact with the target through specific characteristic trajectories. As discussed in Section 2.2, these characteristic trajectories are represented as distinctive *perturbations* of the goal motor state delivered by the reach network, which influence the motor signals generated by the feedback controller. The influence of perturbations on the goal motor state is shown by the “plus” symbol in Figure 3. The perturbation computed by the simple action network is applied to the goal motor state at the start of a reach action and removed when the hand is at a specified distance from the target.

The simple action network takes as input the goal motor state delivered by the reach network, plus a specification of a more complex motor goal: a **motor program**, which in our system is either “grab,” “punch” or “slap”^3^. The output of the network is a perturbation to be applied to the goal motor state. The network starts to learn when the reach network providing its input can reliably generate a goal motor state leading to a touch signal. It is trained to generate perturbations on this goal state that produce trajectories resulting in specific *patterns* of tactile feedback on the hand, corresponding to a grasp, a punch, and a slap. In our current implementation, we define the three distinctive tactile patterns by hand, rather than requiring the system to learn them from scratch; however, since they are quite distinct patterns, there is some prospect for them being learnable using the kind of unsupervised methods discussed in Section 1.2.

To train the simple action network, a simple rigid object (again the cylinder) is presented to the reach network in a random location, generating a visual representation. The reach network computes a goal motor state from this visual representation, which is passed as input to the simple action network, which produces a perturbation of this goal state. This perturbation is annealed with noise, which is progressively reduced to zero during training. The feedback controller moves the hand toward the perturbed goal state; when it attains a certain distance from the target, the perturbation is removed, and the hand approaches the actual goal state. From time to time, the perturbation applied generates a prespecified rich tactile signal. Some perturbations result in a grasp or near-grasp, which generates one class of tactile stimulus. Others result in slapping movements, which generate another, different, class of tactile stimuli, or in punching movements, which generate another distinct class of tactile stimuli (These rich stimuli are almost never generated through pure feedback control, because they result from special trajectories). When a rich tactile stimulus is generated, copy and learning operations take place in the simple action network which are analogous to those in the reach network. First, the tactile stimulus is copied to the area holding “motor programs.” Second, the simple action network is trained to map the current goal motor state, *plus the currently active motor program*, onto the perturbation which resulted in the reward. After this learning, activating a specific motor program will generate an action with a characteristic trajectory, that reliably brings about a particular perceptual effect. We envisage motor programs competing with one another, with a single winner being selected.

In the simple action network, the three motor components of a perturbation are computed one by one, in the three networks labeled N1, N2, and N3 in Figure 5. This is because there are typically several possible perturbations which result in any given tactile reward signal: the network needs to select one of these, and selection of the different components of a perturbation cannot be performed independently. So the network N1 computes the alternative possible values for the first component of the perturbation, then selects one of these, and passes the selected value to the network N2 as input, and N2 performs a similar operation, passing its selected value to N3, which selects the final component.

Each network N1…N3 is a single hidden layer of 15 neurons, fully connected to the input layer that precedes it and to the output layer that follows it, and trained by backpropagation. The output layer of each network is a layer of 10 neurons, holding a place-coded representation of possible perturbations, represented as joint angles. The perturbation angles used to train the network are specified using a coarse-coding scheme. Each angle is assigned to one of 10 “buckets,” which are associated with the 10 neurons; each neuron represents the angle at the mid-point of its assigned range. The neural representation of an angle is generated by strong activation of the neuron corresponding to this “bucket,” and a lesser activation of the two flanking neurons. After training, each network generates a distributed pattern on its output layer. The neuron with the highest activation is used to select a winning coarse-coded perturbation, consisting of the winning unit and the units flanking it on either side. This triplet of activations is converted back to a numerical angle by taking an average of the angles associated with each unit, weighted by their activity. All other units have their activity set to zero before the layer is used as input for the next network.

Note that the simple action network must execute together with the reach network. It modulates the behavior of the simple network, in a manner reminiscent of Brook's (1991) subsumption architecture. In order to execute a simple motor program, it is important that the whole simple action circuit is enabled, or turned on. Accordingly, while different motor programs provide different input to the simple action network, they also uniformly generate a control signal to enable the network they provide input to. This control signal is shown in Figure 3 by the unfilled arrow leading from the simple motor program medium to the simple action network.

#### 2.3.3. The causative action network

**Figure 6:**
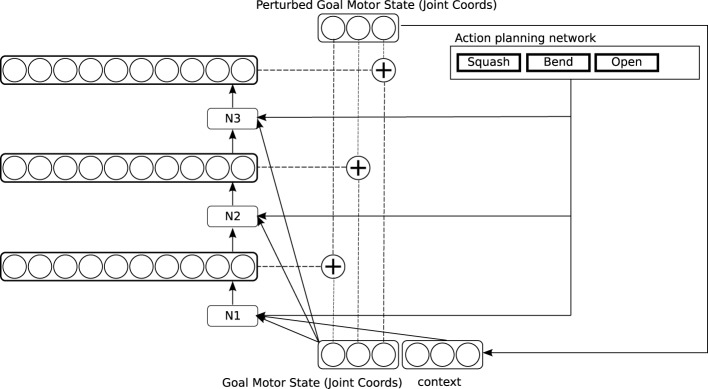
**Detailed architecture of the causative action network**.

The final network to be trained is the causative action network (see the blue part of Figure 3, and Figure 6). This network implements our model of causative actions: it is intended as a model of the ventro-dorsal pathway discussed in Section 1.1. Our key proposal is that above the simple action network there is a higher-level network trained from still more sophisticated sensory signals, which derive not from the tactile system, but from a high-level perceptual module which can classify arbitrary actions taking place in the external world, relying mainly on vision and hearing rather than touch. There is a well-studied perceptual module of this kind in the brain, implemented in a pathway from sensory cortices (in particular visual cortex) through the superior temporal sulcus (STS) and inferior parietal cortex to the “mirror neurons” in ventral premotor cortex (see e.g., Keysers and Perrett, 2004). This pathway also overlaps extensively with the “ventral attentional network,” implicated in allocating attention to salient external events in the world (see e.g., Corbetta and Shulman, 2002). We will refer to these networks jointly as the “action recognition pathway.” This pathway is normally thought of as being engaged when an agent is passively observing the external world. But consider what happens when the agent is attending to an external object *as a target*, while directing the hand toward it along a particular trajectory. Any external actions regularly evoked in the action recognition pathway in this scenario are likely to be actions *caused by the hand's movement*. We propose that during action execution, action signals evoked in the action recognition pathway are hardwired to function as reward signals, which train the causative action network to bring about particular distal actions in the world. The action recognition pathway occupies areas in superior temporal, inferior temporal and inferior frontal cortex that adjoin or overlap with the ventro-temporal pathway (see Section 1.1), so it is certainly well-placed to provide training signals for the causative action network.

Training in the causative action network involves presenting different articulated objects to the system: a lever that can pivot around a joint, and can be bent; a hinged door in a plane, which can be pushed open, and a pair of horizontal plates connected by a spring, which can be “squashed” by pushing down on the top plate (see again Figure 2). Training again proceeds by random generation of perturbations to the goal motor state delivered by the reach network. In this circuit, *sequences* of perturbations are applied, to generate still more complex trajectories (This is depicted in Figure 6 by a recurrent input, though in our implementation we “unroll” this recurrence and generate exactly two perturbations). Some of these sequences cause particular patterns of movement in the target object, which are interpreted as external actions by the action recognition system. Activation of an action representation in the action recognition system when performing an action on a target object is hard-wired to generate a reward signal. This signal has two effects. First, the observed action is copied to a medium in which action plans are held (the action planning system). Second, the causative action network is trained to map the basic goal motor state delivered by the reach network onto the sequence of perturbations which led to reward. Note that the network also takes representations in the action planning system as input. After training, the causative action network can take a simple goal motor state, plus an action representation in the action planning system, and generate a sequence of perturbations which will lead to observation of the planned action on the attended target.

This network enables a rich repertoire of actions to be learned. It preserves Hommel et al.'s idea that action representations are organized around their perceptual effects. But since the action recognition network generates rich, high-level perceptual signals, a correspondingly rich set of motor programs can be established. At the same time, the basic mechanisms through which learning happens are the same as in much simpler motor learning systems.

Part of the design of the causative action circuit is that “cause” is a motor program in its own right, which competes within the motor program selection system against regular motor programs like “grasp” and “slap.” One important difference is that the “cause” action enables the causative actions network rather than the simple action network, but other than that it counts as a regular motor program. This raises some important questions about how causative actions are planned and executed. When an agent decides to perform a causative action, presumably he has some particular caused action in mind. But at the time of planning, this caused action is in the future: minimally, the agent must bring his hand into contact with the target object before he can cause it to move in any way. In order to cause a particular action in a target object, the trajectory of the hand toward the object must often be biased from the very start: for instance, to cause an object to squash, the hand must approach the target from a particular direction, and with particular force (see the discussion in Section 1.2). So the movements which bring about the caused action must be initiated some time before the action is perceived.

Our way of addressing this issue in the network is to activate the motor correlates of perceived actions in a medium holding *planned* actions, rather than in the medium of regular motor programs like “grasp” and “slap.” An underlying assumption in our model, as in many models of motor control, is that the motor system is hierarchical, mapping high-level representations of planned actions and action sequences, predominantly represented in prefrontal cortex, onto lower level action representations in premotor and motor cortices (see e.g., Miller and Cohen, 2001; Averbeck et al., 2002; Saito et al., 2005). In our model, prefrontal cortex stores planned sequences of attentional and motor operations (Knott, 2012), which can be activated from visual inputs, through the “object classification” pathway in ventral/inferotemporal cortex. On this account, prefrontal cortex is an extension of the ventral object classification pathway; see Cagliore (Cagliore et al., 2010; Thilla et al., 2013) for other expressions of this idea. We call the network that links the ventral object classification system to the prefrontal action planning system the **plan activation network** (Our implementation of this network is very simple, as we do not compute detailed representations of object shape; the model of Cagliore et al. is more detailed, and our model should really be thought of as a stand-in for a more detailed model of this kind). Note that since the ventral object classification pathway computes “semantic” representations of object classes, the plan activation network learns quite high-level functional representations of objects as tools: the motor affordances generated in this network are “stable” compared with those computed directly from visual representations in the dorso-dorsal pathway (see e.g., Borghi and Riggio, 2009 for relevant discussion). We assume that planned sequences in prefrontal cortex are selected as wholes, and that the component actions in a planned action sequence are active in parallel, even if they are executed in sequence (This assumption is well-supported by single-cell recordings in monkeys; see e.g., Averbeck et al., 2002). When the causative actions network is exploring causative actions, it will activate the “cause” motor program experimentally, and choose a random sequence of perturbations. In some cases, this results *some time later* in activation of an action in the action recognition system: say “squash.” This observed action activates a corresponding planned action. Additionally the plan activation network (see the bottom right of Figure 3) learns that the sequence “cause,” “squash” produces observable effects on the category of object currently present, so that when a similar object is presented in future, it will tend to activate this planned sequence as a possible plan in the action planning system, to compete against other possible plans^4^. Now consider what happens when the planned sequence is executed. The agent first executes the motor program “cause.” This enables the causative action network, which generates a sequence of perturbations. Crucially, the causative action network also takes input from the planning medium in which the caused action (“squash”) is active as part of the planned sequence. So as soon as it is initiated, the network is configured to generate the perturbation sequence which led to the caused action, even before this action actually occurs^5^

The key mechanism enabling causative actions to be executed is one which activates a sensory representation (the squash action) *as a goal* some time before it is evoked as a sensory stimulus. Note that something very similar happens in the other networks; for instance in the reach network the actual motor state where the touch sensation occurs is activated as a goal motor state. In the simple network this activation is possible because visual perception provides information about reward-associated motor states. In the higher-level causative actions network, the advance notification of reward comes from the working memory system which stores prepared actions. But the effect is much the same.

## 3. Evaluation of the model

The reach network performs well after training; its performance is described in Lee-Hand et al. (2012); in this section we discuss the performance of the simple action and causative action networks after training.

**Figure 7:**
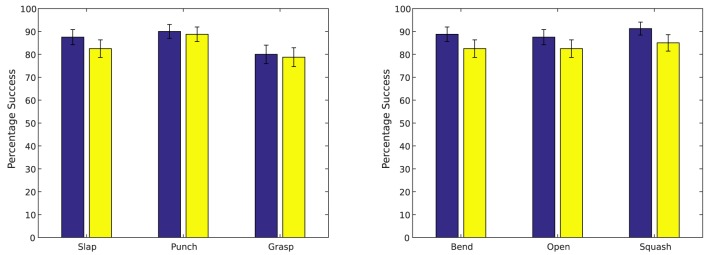
**Left:** Results from testing the simple action network. **Right**: Results from testing the causative action network. Results are averaged across 10 trials of 8 object locations; results for seen locations are shown in blue; results for unseen locations in red. Error bars show standard deviation.

The trained simple action network was tested by presenting a cylinder at the locations seen during training (**Figure 9A**) and at a number of new locations (**Figure 9B**), activating a simple motor program at random (grasp, slap or punch), and observing how often the tactile stimulus associated with this motor program was produced. Results of these tests are summarized in Figure 7 (left). The trained causative action network was tested by presenting one of the articulated objects at the seen and unseen locations shown in Figure 9, and observing how often the network generated a series of perturbations that led to the action perception system registering the action appropriate for the object. Results of these tests are presented in Figure 7 (right).

**Figure 8:**
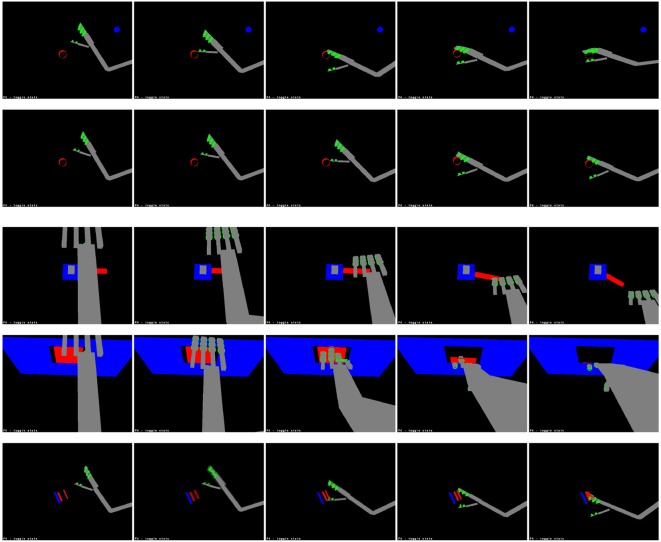
**Learned actions**. From top: grasping, slapping and punching a cylinder; bending a lever, opening a door and squashing a sprung plate. These sequences are taken from the latter stages of each action, when the hand makes contact with the target.

In general, the system was quite successful in producing motor actions with the expected perceptual consequences. The simple action network produced actions resulting in the expected tactile stimuli for an average of 86.25% of seen target locations and an average of 81.2% of unseen locations; the causative action network produced actions resulting in the target undergoing the expected action in an average of 86.7% of seen locations and an average of 83.8% of unseen locations. The difference between seen and unseen locations was statistically significant for both types of action (*p* = 0.016 for simple actions and *p* = 0.001 for causative actions, by unpaired one-sided *t*-tests); the difference between simple and causative actions was also significant (*p* = 0.04, by an unpaired two-sided *t*-test). Illustrations of representative successful actions of each type are shown in Figure 8.

**Figure 9:**
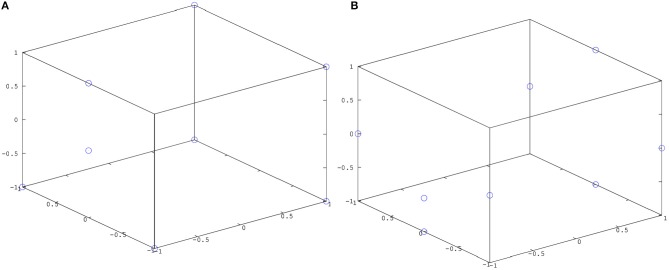
**Possible locations of target objects for the simple action and causative action networks (A) during training; (B) during testing**.

The cases where actions do not result in the expected sensory consequences can be accounted for by two main factors. Most failures are due to the simplicity of the feedback motor controller that moves the hand toward a goal state. The hand/arm system is subject to complex Coriolis forces when in motion, and there are limits to how precisely it can be controlled by a simple feedback controller. A few failures result from difficulties generalizing from training locations to unseen locations, but in general the networks do this quite well.

## 4. Discussion

As discussed in Section 1.2, there are many computational models of how agents learn about the perceptual effects of their actions. A novelty of our model is its proposal that there are three qualitatively different *types* of effect-based motor action, that are implemented in distinct neural circuits, which are separately learned one by one. The first two types of action (reaching-to-touch, then actions involving distinctive hand preshapes such as grasping, punching and slapping) have been widely studied, and the idea that they involve distinct circuits is also widely accepted (see e.g., Jeannerod, 1996). Our model is novel in characterizing these actions uniformly in terms of their perceptual effects, in particular their tactile effects. However, the main novelty in our model is the proposal that there is a third type of effect-based action, which produces effects that are perceived as independent events in the world. Existing computational models are certainly able to learn *instances* of actions of this kind—for instance the model of Katz and Brock (2008) learns actions causing changes in the configuration of articulated objects, and those of Stoytchev (2008) and Ugur et al. (2011) learn actions causing changes in the position of moveable objects. But our model is novel in treating these actions *as a class*, distinct from simpler effect-based actions. Since our causative actions network is trained by the same module that identifies the actions of independent agents in the world, it subsumes a large and diverse set of causative actions under a single mechanism: thus, for instance, it proposes that the same mechanism that learns how to move an object in space is also the mechanism that learns how to make an object deform, or for that matter, walk, or talk, or dance. We have demonstrated this principle by showing that the same mechanism can learn three quite different causative actions, on three target objects with quite different patterns of articulation. No other model of motor learning can show this performance, to our knowledge.

We conclude by considering how our proposed class of causative actions might be extended and identified experimentally, and discussing some uses to which it might be put.

### 4.1. Causative actions that do not involve physical contact

In our model, causative actions always bring about effects on the target object by physical touch. But in the real world, actions can also have causative effects through less direct means: for instance, an agent can cause a feather to move by blowing it, or cause another agent to cheer up by smiling at him/her. Since our causative actions network is trained by a system that perceives distal events in the world, it is certainly capable of learning actions that achieve causative effects on objects without making direct contact with them. The key requirement for this learning is the same as that discussed in Section 2.3.3: the agent must be *attending to the target object* when performing the causative action. Since spatial attention actions are initially learned through associations with physical touch, this attentional action will ensure that the causative action is directed *toward* a certain object, even if in this case the object is not touched. Crucially, it also ensures that the agent's action perception system is directed at this same object, so any effects on it are detected.

For “social” actions such as cheering someone up, focal attention on the target object is typically necessary to achieve causative effects, but may not always be sufficient. The agent to be influenced must also typically *return* attention, so that “joint attention” is achieved (Tomasello, 2003). It may be that for social actions, the sensation of an attended agent returning one's gaze plays a role similar to that played by the sensation of touch for physically causative actions, in establishing the conditions under which causative actions can take place. The stimulus of direct gaze certainly modulates cognitive processing in powerful ways, similarly to the stimulus of touch (Senju and Johnson, 2008). This is an idea we intend to explore in future research.

### 4.2. Experimental predictions about causative and non-causative actions

We have already suggested in Section 1.1 that our distinction between causative and non-causative actions coincides with the distinction made in experimental work between actions on tools implemented in the ventro-dorsal pathway and “volumetric” actions implemented in the dorso-dorsal pathway. As discussed in that section, the examples of “functional” actions used in the experiments of Bub et al. (2008) and Jax and Buxbaum (2010) are mostly causative actions in our terms (i.e., they cause a movement in the target object which constitutes an event in its own right), and the examples of “volumetric” actions are mostly non-causative in our terms (i.e., they do not cause such a movement). However, there are cases where the experimental action stimuli do not conform to these definitions: for instance, grasping a mug by its handle is classed as functional, but it is not causative in our terms. We predict that these actions will be outliers in their groups in the above experiments, and that the experimentally observed distinctions between functional and volumetric actions will be sharper if they are removed, or possibly reclassified. We also predict more generally that *any* action that is non-causative in our terms will pattern with “volumetric” actions, and evoke activity in the dorso-dorsal pathway—including actions like punching and slapping—and that any action that is causative in our terms will pattern with “functional” actions, and evoke activity in the ventro-dorsal pathway. These are general predictions that could certainly be tested in further experiments^6^.

Note that our model can already account for much of the evidence for effect-based motor representations discussed in Section 1.1. All the experiments described in that review in fact involve actions which in our definition would be classed as causative—that is, actions that bring about independently observable events—the illumination of a light in Hommel (1993), the opening or closing of a pair of tongs in Umiltà et al., 2008), the occurrence of a tone in Elsner et al. (Elsner and Hommel, 2001; Elsner et al., 2002). Consider for instance Umiltà et al.'s demonstration that many F5 neurons in macaque encode the perceived effects of motor actions on a pair of held tongs (their opening or closing) rather than motor actions themselves. Opening and closing are causative actions: to open *X* is to cause *X* to open. In our model, the event of the tongs opening (or closing) will be present in the action planning system (shown in blue in Figure 3) regardless of the motor action that brings it about. If we assume that the action planning system is partly implemented in F5, the invariant responses observed by Umiltà et al. in F5 are expected (Note that F5 is indeed a component of the ventro-dorsal motor pathway; see e.g., Rizzolatti and Matelli, 2003). To take another example: consider the ideomotor response documented by Elsner and Hommel (2001): in subjects who have learned that an certain action generates a certain tone, activation of this tone by itself as a perceptual stimulus can prime execution of this action. Note that perception of the tone in this scenario does not actually *trigger* the action; it just activates it at a sub-threshold level. In our model, the link between a perceived event and the planning representation responsible for bringing it about is normally gated shut (see again the blue part of Figure 3); it is only gated open after the agent has actually executed an action, and has perceived an event that the action might have caused. To account for the ideomotor response, we can simply assume that the link from perceived events to planned actions can never be fully closed: that some activation always gets through. On this account, the ideomotor response is a side-effect of the circuit that supports learning of causative actions, as proposed in Section 1.3. One final interesting prediction addresses the relationship between the ventro-dorsal “use” motor pathway and the action recognition pathway, which occupy adjacent, and possibly overlapping, areas of superior temporal, inferior parietal and inferior premotor cortex. We suggest these pathways can be distinguished by contrasting brain activity in two conditions: one in which an agent executes a causative action bringing about a certain movement in the target object (e.g., closing a door), and another in which the agent observes the same movement occurring in the target object by itself without the causative action (i.e., observing “the door closing”). We predict that the part of ventro-dorsal cortex involved in representing the caused action will be invariant across these conditions, while the part representing the causative action will be active in the first condition but not the second. This is a prediction that could be tested, for instance in an imaging experiment.

### 4.3. Causative actions in a model of action perception

Our model of causative actions has interesting implications for an account of action perception. A common idea is that perceiving an action performed by someone else involves activating the same motor representations that are active when we perform it ourselves (see e.g., Rizzolatti et al., 2000). This is often called into question (see e.g., Hickok, 2008). But it is interesting that the debate often turns on actions that are causative, in our sense. For instance Hickok considers the action of *playing the saxophone*: how can an observer who cannot execute this action recognize someone else doing so, if action recognition involves activation of representations in one's own motor system? To us it is relevant that “to play the saxophone” is to *cause the saxophone to play*. Minimally, this means that the kind of “motor resonance” that we expect during perception of someone else playing the saxophone will be different from that associated with perception of simple actions like touching or grasping. Exactly what form this resonance might take is a matter for further thought: there is no ready-made incarnation of the mirror system hypothesis configured to causative actions. But if playing the saxophone involves perceiving “the saxophone playing” as an independent event, it is likely that perceiving *someone else* playing the saxophone involves exactly the same perceptual representation of this event. So our model certainly predicts some overlaps between the representations involved in executing and perceiving this action. We suggest that the best way to extend our model to support perception of causative actions would be to include a general perceptual mechanism for identifying causally related events in the world (see e.g., Scholl and Tremoulet, 2000), so that the observer can identify not only that the saxophone is playing, but that the observed agent's motor actions are causing this. This model would preserve a close parallelism between action execution and action perception, as posited by the mirror system hypothesis, without requiring that the observed agent's movements resonate in detail with motor programs of the observer.

### 4.4. Simple actions with causative effects

While we have construed slapping and punching as simple actions, an agent can also slap a cup *across the table*, or punch a person *to the ground*. The existence of such actions may be thought to cast doubt on the taxonomy of effect-based action types we are proposing: they are clearly causative actions, in that they bring about episodes involving the target object, but they are also simple actions. In fact there is no reason why an action that brings about an independent episode cannot also be describable as a simple action. In fact this possibility enables an elegant model of the way actions are described linguistically. We can refer purely to the causative components of an action, by saying e.g., that the agent *moves* the cup across the table. We can also refer purely to an action's identity as a simple action, by saying e.g., that the agent *slaps* the cup (without mentioning the effect of this action on the cup). Or, most informatively, we can refer to both the simple and the caused effect, by saying that the agent slaps the cup across the table.

### 4.5. Causative actions in language and the motor system

Our model does not include an account of how perceptual and motor representations interface with language, but there are some extensions that could readily be made in this direction. In particular, we could assume that common nouns denoting tools directly activate object representations in the ventral pathway mapping object categories to the sets of “functional” actions they afford, an assumption which is common in the literature (see e.g., Pulvermüller et al., 1999; Bub et al., 2008; Cagliore et al., 2010); and we could assume that action verbs directly activate motor programmes in the relevant simple-action and causative-action networks (see e.g., Hauk et al., 2004). With these assumptions, our model's behavior would be consistent with a number of recent experiments relating to motor priming. Masson et al. (2008) showed that in sentences that name tools but not associated motor actions (e.g., *the scientist looked at the stapler*), tool nouns primed functional actions but not reach/grasp actions. If tool nouns activate units in the plan activation network in our model, this will result in activity in the causative action network but not the reach or simple-action network. Bub et al. (2008) showed that tool words generate activity in the “use” pathway earlier than the reach/grasp pathway. If tool nouns directly activate “use” actions in the action planning system in our model, it will show the same effect, since the link from tool nouns to actions in the simple action network runs through low-level visual representations, and so is much less direct.

Our distinction between causative and non-causative actions also relates closely to a distinction made by theoretical linguists between the *syntactic* structures associated with particular verbs. Our test for causative actions in fact identifies a well-studied class of verbs: those that undergo the so-called “causative alternation” [see e.g., Schaefer (Schäfer, 2009) for a review]. The causative alternation is illustrated in the following two sentences:

(1) John bent the lever.
(2) The lever bent.

These sentences are interesting for linguists because the lever appears as the subject of Sentence (4.5) and the object of Sentence (4.5). This is puzzling because as a rule we expect the semantic roles associated with the noun phrases in a sentence to relate to their syntactic roles. To allow generalizations about the semantic contributions of subjects and objects, linguists have often proposed that the syntactic structure of the transitive sentence *John bent the lever* involves a “hidden” cause predicate, as illustrated below (see e.g., Levin Levin and Rappaport Hovav, 1995):

(3) John caused [the lever bent].

While this proposal is made on purely syntactic grounds, it clearly echoes the motor model of causative actions introduced in the current paper. It is interesting to speculate that this is no coincidence—that the “cause” predicate posited by linguists refers to some representation engaged by the causative actions pathway in the motor system. This would be evidence for the kind of “embodied” model of syntax proposed by several theorists [see e.g., Feldman and Narayanan, 2004, Zwaan et al. (Zwaan and Taylor, 2006); Knott, 2012]. The idea that causative sentence structures engage the causative motor actions pathway could be tested in several ways: for instance it predicts that sentences whose structure contains an implicit cause predicate like *John bent the lever* activate the ventro-dorsal pathway, while superficially similar sentences like *John touched the lever* or *John slapped the table* activate the dorso-dorsal pathway. If such predictions are borne out, the proposal that the dorso-dorsal pathway implements causative motor actions will shed some interesting light on the general question of how natural language syntax relates to the motor system.

## Funding

This work was supported by the New Zealand Marsden Foundation, Grant 13-UOO-048.

### Conflict of interest statement

The authors declare that the research was conducted in the absence of any commercial or financial relationships that could be construed as a potential conflict of interest.

## Figures and Tables

**Algorithm 1:**
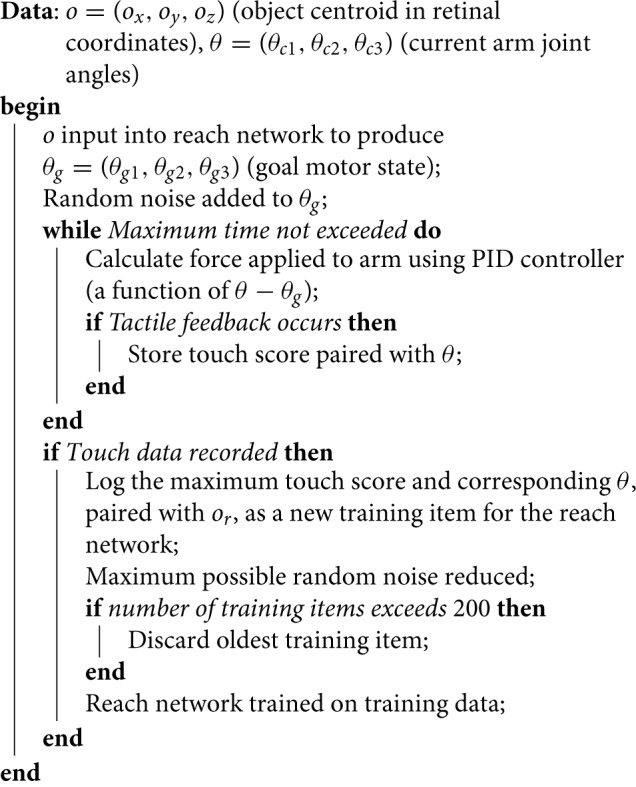
Learning Goal Arm states for reach actions

**Algorithm 2:**
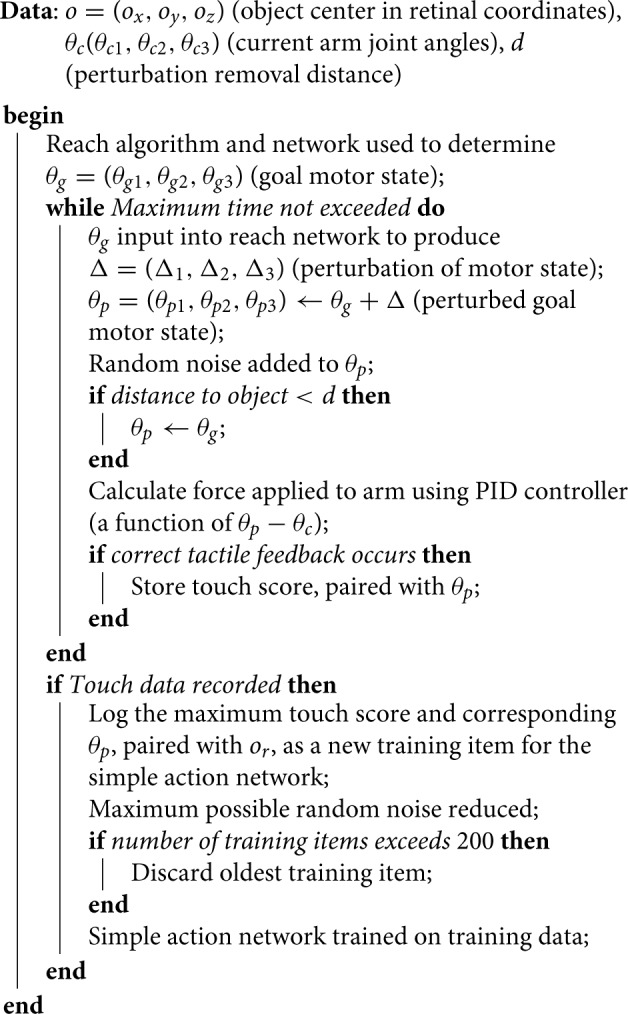
Learning simple motor programs.

**Algorithm 3:**
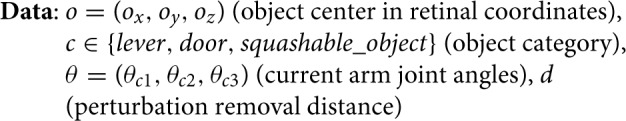
Learning causative actions.

**Algorithm 4:**
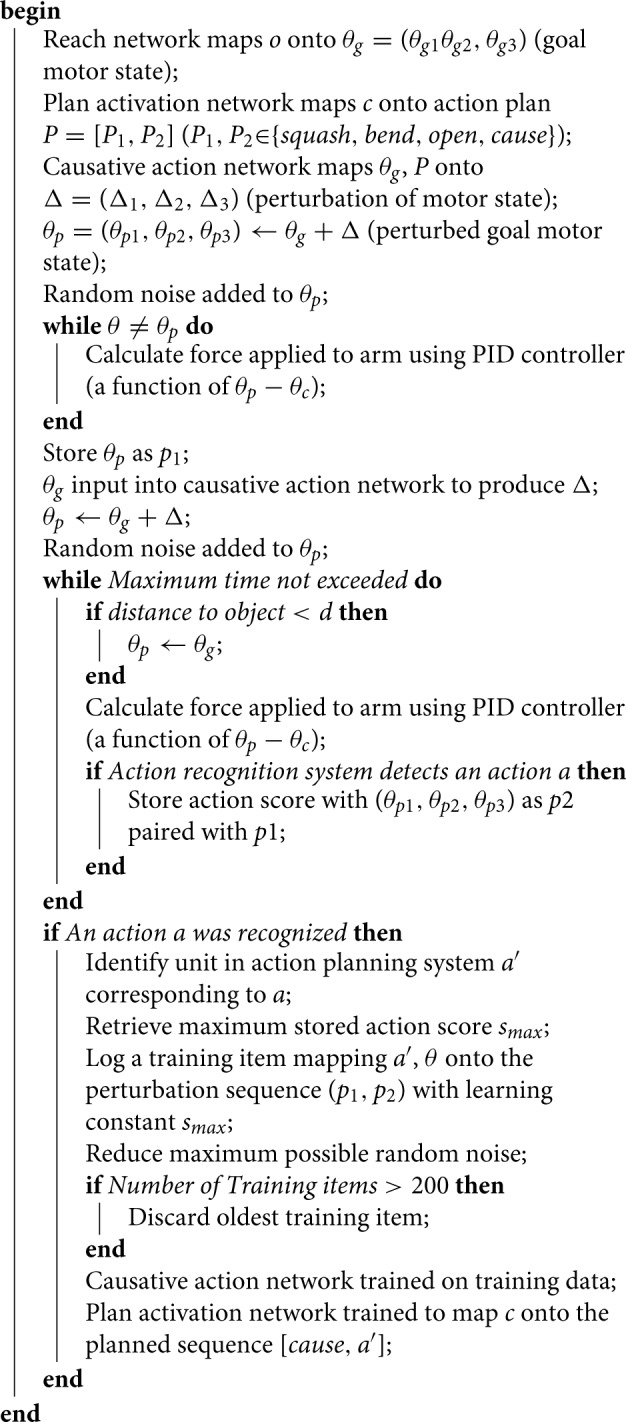
Learning causative actions.

## Footnotes

^1^Note that the ventro-dorsal pathway computes its own volumetric representations of objects, which supplement the high-level functional affordances associated with the category of the target object with lower-level information about its size, shape, and orientation (Area AIP has an important role in computing these representations; see e.g., Murata et al., 2000).

^2^We should note that while effect-based action representations play a useful role in accounts of how agents explicitly *simulate* actions and their consequences, we will not be addressing this question in the current paper, which focusses on the mechanisms involved in actual execution of actions. We discuss simulation processes in other work (Takac and Knott, 2013, 2015).

^3^In Figure 3 we specify a third input, a representation of “volumetric” target shape that is also computed by the dorso-dorsal pathway; however in our current system this is unimplemented.

^4^In the current paper we do not model the process by which alternative possible plans compete; this process is the focus of another model (see Takac and Knott, 2013, 2015).

^5^In Figure 3, the causative action network also takes input from a region representing object shape, that provides the kind of “volumetric” information computed in the early stages of the ventro-dorsal pathway, especially in AIP (as discussed in Section 1.1). Again, since we do not vary the size or orientation of our target objects, objects, we do not yet implement this input.

^6^There are a few caveats to note. Firstly, some causative actions are achieved through movements that also happen to be in the repertoire of non-causative actions: for instance, if an agent punches someone *to the ground*, they are causing that person to go “to the ground” through an action that also happens to be defined as a simple action. In this case, we expect activity in both dorso-dorsal and ventro-dorsal pathways. Secondly, some non-causative actions are *preparatory* to a subsequent causative action: for instance grasping a mug by its handle is often preparatory to lifting it or tilting it (i.e., causing it to lift/tilt). In these circumstances, there may again be activity in both pathways.

## Appendix

### Training algorithms

In this Appendix we present the training algorithms for the reach, simple action and causative action networks in more detail.

The first network that is trained is the reach network. A single object (the cylinder) is presented in each training trial in any location within the space reachable by the arm. The retinotopic location of this object is computed, and provided as input to the reach network, which generates an output goal motor state. Initially this output is annealed with noise, so the goal motor state is essentially random. A feedback controller then brings the hand toward the goal motor state. If the hand happens to make contact with the object (i.e., if a tactile signal is received), the current motor state is logged as training data for the reach network, paired with the retinotopic location of the object. After each trial, the reach network is trained on all the training data logged so far. During learning, the noise applied to the network's output is progressively reduced to zero. Pseudocode for training is shown in Algorithm 1.

There is one further point to make about the training of the reach network (and those that follow). It is of course unrealistic to assume a storage medium where a large number of training items can be logged. In a more plausible online learning scheme, the network being trained would interleave self-generated pseudo-training items with new training data arriving sequentially (see e.g., Robins, 1995). To approximate an online scheme, we do however impose various constraints to ensure that the quality of stored training data improves during the course of training. Firstly, the learning rate used by the network for any given logged training item is a function of a **score** associated with the haptic feedback pattern that the hand received (recall some patterns are better than others). This means that trajectories which result in higher scores influence the behavior of the network more than trajectories producing lower scores. In addition to this, there is a minimum threshold score needed in order for an action to be logged as training data, which is increased as learning proceeds. Finally, we only retain the most recent 200 logged training items to use for training.

The next network to be trained is the simple action network. In each training trial a single object (the cylinder) is presented in one of a small number of training positions, as shown in Figure 9A. The trained reach network generates a goal motor state as usual; this is passed as input to the simple action network, along with a randomly selected simple action category (grasp, slap or punch). The output of the network is a perturbation, which is applied to the goal motor state. This output is again annealed with noise, which is reduced to zero as training progresses. The motor controller then moves the hand in the direction of the perturbed goal motor state, and when the perturbation is removed, in the direction of the actual goal motor state. If the resulting movement activates one of the predefined classes of rich tactile signal, a training item is logged, mapping the actual goal motor state providing input to the network, plus the simple action category corresponding to the activated tactile signal, onto the perturbation that was applied. After each trial, the network is trained on all the logged training items. This training algorithm is shown in Algorithm 2.

Finally the causative action network is trained. In each training trial either the squashable, bendable or openable object is presented in one of the locations given in Figure 9A. The plan activation network maps the object's category onto a planned sequence of two actions (a pair of two actions in the action planning system). The causative action network maps the the current goal motor state and the planned action sequence onto a sequence of two perturbations (These outputs are again annealed with noise, reduced to zero during training). The two perturbations drive the hand/arm along a particular trajectory. If this trajectory happens to result in the target object undergoing an action (bend, open or squash), the action recognition system will activate the relevant action category, and a training item is logged, mapping the current goal motor state, plus a unit in the action planning system corresponding to the recognized action category, onto the perturbation sequence. At the same time, the plan activation network learns to map the category of the target object onto the sequence “cause,” followed by the perceived action.

This training regime is detailed in Algorithm 3.
